# The effects of intraovarian injection of autologous menstrual blood-derived mesenchymal stromal cells on pregnancy outcomes in women with poor ovarian response

**DOI:** 10.1186/s13287-023-03568-1

**Published:** 2023-11-15

**Authors:** Simin Zafardoust, Somaieh Kazemnejad, Mina Fathi-Kazerooni, Maryam Darzi, Mohammad Reza Sadeghi, Ali Sadeghi Tabar, Zahra Sehat

**Affiliations:** 1 Nanobiotechnology Research Center, Avicenna Research Institute, ACECR, Tehran, Iran; 2 Monoclonal Antibody Research Center, Avicenna Research Institute, ACECR, Tehran, Iran; 3 Reproductive Biotechnology Research Center, Avicenna Research Institute, ACECR, Tehran, Iran; 4 Avicenna Fertility Clinic, Avicenna Research Institute, ACECR, Tehran, Iran

**Keywords:** Poor ovarian responder, Menstrual blood stromal cells, Cell therapy, Clinical trial, Infertility

## Abstract

**Background:**

Assisted reproduction faces a significant obstacle in the form of poor ovarian response (POR) to controlled ovarian stimulation. To address this challenge, mesenchymal stem cell therapy has been proposed as a potential treatment for female infertility and/or restoration of ovarian function in POR women. Our previous research has demonstrated that menstrual blood-derived-mesenchymal stromal cells (MenSCs) injected into the ovaries of women with POR can increase pregnancy rates. The objective of this study was to examine whether MenSC therapy could enhance ovarian reserve parameters and pregnancy outcomes in a larger population of individuals with POR.

**Method:**

This study consisted of 180 infertile individuals with POR who declined oocyte donation. Participants were divided into two groups: those who received bilateral MenSCs intraovarian injection and those who received no intervention. Our primary aim was to compare the rates of spontaneous pregnancy between the two groups, followed by an investigation of any alterations in the ovarian reserve parameters, such as serum FSH, AMH, and AFC levels, as well as the ICSI/IVF outcomes, in both groups of participants.

**Results:**

The MenSC therapy exhibited a favourable tolerability profile and did not raise any safety concerns. Following the 2-month follow-up period, women who received MenSC treatment demonstrated a significantly higher rate of spontaneous pregnancy (*P* < 0.005) and an improvement in anti-Müllerian hormone (AMH) levels (*P* = 0.0007) and antral follicle count (AFC) (*P* < 0.001), whereas the control group demonstrated a considerable decline in these parameters (Both* P* < 0.001). The MenSC therapy led to a greater number of mature oocytes and embryos among women who underwent ICSI/IVF. Our age subgroup analysis demonstrated a significant difference in the number of spontaneous pregnancies and ICSI/IVF outcomes between the treatment and control groups only among individuals below 40 years of age.

**Conclusion:**

The results of our study indicate that MenSCs treatment may be a viable option for treating women experiencing POR. However, in order to be widely implemented in clinical practice, the clinical effectiveness of MenSCs therapy will need to be established through rigorous prospective randomized clinical trials.

*Trial registration*: ClinicalTrials.gov Identifier: NCT05703308. Registered 01/26/2023, retrospectively registered, https://clinicaltrials.gov/ct2/show/NCT05703308. IRCT, IRCT20180619040147N4. Registered 08/01/2020.

## Introduction

Nowadays, more women seek infertility therapy due to economic development's impact on reproductive delays [1]. Despite developments in assisted reproductive technology (ART), treating infertility in women with poor ovarian response (POR) remains a daily challenge for doctors since this population usually experiences menstrual abnormalities, ovulation disorders, infertility, and organ and systemic declines linked to low estrogen levels [2, 3]. According to the European Society for Human Reproduction and Infertility (ESHRE) standard, two of the following criteria are necessary to diagnose POR: (i) advanced maternal age (≥40 years old) or any other POR risk factor; (ii) a previous cycle with three oocytes retrieved using conventional stimulation; and (iii) an abnormal ovarian reserve test (i.e. antral follicular count (AFC), <5–7 follicles, or anti-Müllerian hormone (AMH), <0.5–1.1 ng/ml) [4].

POR accounts for 9–24% of controlled cycles of in vitro fertilization (IVF) [5], which is also linked to high cycle cancellation rates, low oocyte maturation rates, and low pregnancy rates [6, 7]. In most cases, the regular brief protocol and moderate stimulation protocol are used to treat POR patients [8]. However, none of these regimens significantly improved IVF outcomes. As a result, more innovative approaches to treating POR infertility and preventing women from donating their oocytes and embryos are needed.

Recently, with the advent of regenerative medicine, several preclinical studies have been conducted to assess the safety and efficacy of stem cell therapy in treating female reproductive disorders [9–14]. Adult mesenchymal stromal cell (MSC) therapy has gained particular interest because it may provide a supportive microenvironment for oocyte development from quiescent primordial follicles [14]. These stem cells can migrate to damaged tissue and release cytokines and growth factors that support angiogenesis, anti-apoptosis, and anti-fibrosis to repair the ovary [15, 16]. In preclinical studies, human MSC transplantation hosted the ovaries and restored their function and structure in premature ovarian failure (POF) animal models [14, 17]. Growth factors produced by these cells, including IGF-1 (Insulin-like growth factor I), TGF-ß (Transforming growth factor ß), FGF1 and FGF2 (Fibroblast Growth factors 1 and 2), and EGF (Epidermal Growth Factor), have previously been reported to be associated with ovarian folliculogenesis [18].

An essential point is that in patients with diminished ovarian reserve (DOR), the ovary contains residual dormant primordial follicles that theoretically might be activated by stem cell therapy. In fact, Herraiz et al. [19] reported on the benefits of stem cell treatment in a premature ovarian insufficiency (POI) mouse model. Fertility rescue and spontaneous pregnancies were achieved after human bone marrow-derived mesenchymal stem cell (BMSC) infusion in a mouse model of ovarian insufficiency. In this study, when human BMSCs were infused into mice xenografted with human ovarian cortex from POR patients, the injected cells were engrafted close to vessels and granulosa cells, which promoted follicular growth to the secondary stage. They also demonstrated that BMSC infusion increased ovarian stromal proliferation and blood vessel formation while decreasing apoptosis in both murine and human xenografted tissue. Following these remarkable results, Herraiz et al. conducted the first clinical study to assess the effects of autologous BMSC ovarian perfusion on ovarian reserve and IVF outcomes in women with POR. There have been reports of significant improvements in ovarian function and successful pregnancies after cell transplantation. They found that cell therapy can enhance follicle growth and motility through the secretion of FGF2 and thrombospondin-1 [20, 21]. As a result of these positive results, several clinical studies have been conducted using various sources of MSCs to treat infertility [9, 22–29].

In recent years, one of the favourite sources for researchers to access stem cells has been endometrium. It was discovered in 2004 that the basal layer of the endometrium contains clonogenic stromal cells that proliferate on a regular and dynamic basis. The phenotype, self-renewal capacity, transdifferentiating capacity, and expression pattern of various cell surface antigens of these cells endow them with biological properties comparable to stromal-like MSCs [30, 31]. Since endometrial stem cells must be removed through invasive surgery, the highly proliferative menstrual blood-derived mesenchymal stem/stromal (MenSCs) have received much attention [32]. The potential of MenSCs is due to their advantageous features, such as ease of access, high availability, the possibility of monthly sampling, lack of ethical concerns, absence of tumorigenic potential, secured properties, considerable capacity for trans-differentiation, and originating from endometrial tissue, which make them particularly useful in treating reproductive disorders [30, 31, 33].

Zheng et al., for first time in 2018, speculate that MenSCs could be an effective medical tool for repairing and regenerating reproductive system cells and tissues [34]. MenSCs may be crucial for proper ovarian and endometrial functions, embryo implantation, and the successful progression of pregnancy because they have been shown to have vascular remodelling and immunoregulatory roles by suppressing the expression of inflammatory factors and encouraging type 2 T helper cytokines (Th2) like interleukin-4 (IL-4) [35].

Based on this evidence, we attempted to assess the safety and efficacy of intraovarian injection of MenSCs for the treatment of infertility in POR patients in previous study [36]. In this clinical trial, phases I–II, in 15 women defined as POR using the ESHRE criteria, MenSCs were delivered directly to one ovary for each patient in an effort to optimize the recruitment of existing dormant follicles to improve ovarian reserve markers and pregnancy outcomes. Four of the 15 patients in the cell-treated group became pregnant naturally during the first 3 months post transplantation. Furthermore, three clinical pregnancies occurred in other patients that underwent intracytoplasmic sperm injection (ICSI) cycles, which finally led to five live births in the main group. In addition, raise of AFC and oocyte numbers in comparison with the previous cycle was significant. Nonetheless, the oocyte fertilization rate and embryo number in MenSC group were higher than in the control group. Therefore, we have designed a Phase III clinical study using autologous MenSCs treatment for POR patients based on these findings. This study aims to assess how MenSCs injection affects pregnancy outcomes.

## Materials and methods

### Study design and patient selection

The present clinical trial was a non-randomized, open-label, parallel-group study conducted between June 21, 2020, and September 22, 2021, at Avicenna Fertility Clinic in Tehran, Iran. The study consisted of 180 women with a history of POR who provided informed consent after receiving a detailed explanation of the procedure from the physicians. The data and safety monitoring board of the Avicenna Research Institute closely monitored and analysed the data during the trial. The study was approved by the Biomedical Research Ethics Committee of the Academic Center for Education, Culture, and Research (ACECR) and recorded in the Iranian Registry of Clinical Trials (IRCT20180619040147N4).

The enrolled patients with POR had a history of at least one standard ICSI cycle with an oocyte number less than four and AMH levels lower than 1.1 ng/ml. Table 1 outlines the inclusion and exclusion criteria for this study. The eligible participants (*n* = 180) were allocated to either the MenSCs therapy group (*n* = 90) or the control group (routine ICSI plan, *n* = 90). Patients in the treatment group were monitored for spontaneous pregnancy for 2 months following stem cell transplantation, whereas patients in the control group were followed up 2 months after their last ovarian stimulation for ICSI or IVF. In the cell therapy group, if no pregnancy was reported during the first 2 months after the injection, the patient entered the ICSI/IVF cycle. Ovarian reserve tests were performed on the second or third day of the menstrual period before cell injection and 2 months later. The pregnant participants were closely monitored throughout the conception period, and any information on pregnancy complications and foetal health was meticulously documented.

**Table 1:** Inclusion and exclusion criteria used to select participants for the clinical trial

Inclusion criteria	Exclusion criteria
Written informed consent	Positive history of hydrosalpinx or anatomical uterine disorders (In vaginal sonography or HSG)
Age between 25 and 45 years	Severe male factor infertility of their husbands (count < 15 million/ml)
Serum AMH < 1.1 ng/ml (at the screening visit and in the absence of OC or sex-steroid intake)	
Antral follicular count (AFC) in both ovaries < 4 (at screening visit and in the absence of OC or sex-steroid intake)	
Positive history of at least1 standard previous IVF-ET or ICSI-ET	
Normal thyroid hormones (TSH and FT4)	
Normal level of prolactin	
Normal level of fasting blood sugar	
Normal Liver tests (SGOT, SGPT)	
Normal level of BUN, creatinine	
Negative Infectious tests (HIV, HCV, HBS Ag, VDRL)	
Normal coagulation factors (PT, PTT, BT)	
Normal serum levels of sodium, potassium, calcium, phosphorus	
Negative history of endometrioma or other ovarian cysts	
Negative history of previous ovarian surgery	
Negative history of cancer	
Negative history of a known autoimmune disorder	

*AMH*: anti-Müllerian hormone; *IVF-ET*: in vitro fertilization-embryo transfer; *ICSI-ET*: intracytoplasmic sperm injection-embryo transfer; *TSH*: thyroid stimulating hormone; *FT4*: free T4; *SGOT*: serum glutamic oxaloacetic transaminase; *SGPT*: serum glutamic pyruvic transaminase; *BUN*: blood urea nitrogen; *HIV*: human immunodeficiency virus, *HCV*: Hepatitis C virus, *HBS Ag*: hepatitis B surface antigen; *VDRL*: venereal disease research laboratory test; *PT*: prothrombin time; *PTT*: partial thromboplastin time; *BT*: bleeding time

### Collection and preparation of MenSCs

The group undergoing treatment with MenSCs had their menstrual blood collected using sterile menstrual cups (Diva International Co., Lunette, Finland) on the second day of menstruation. An ultrasound (Honda 2000–5 MHz, Japan) was used to measure the number of antral follicles. Following this, the samples were placed in collection tubes containing Gibco Dulbecco Modified Eagle Medium-F12 (DMEM-F12), 100 g/mL streptomycin, 2.5 mg/mL fungizone, 100 U/mL penicillin, and 0.5 mg/mL EDTA in Good Manufacturing Practices (GMP)-grade phosphate-buffered saline (PBS) without Ca^2+^ or Mg^2+^. The samples were then transferred immediately to a class B cleanroom for MSC isolation and culture under PICS/GMP and ISO9001 regulations, as described in a previous publication [34]. The samples were suspended in DMEM-F12 medium, supplemented with 5% HyClone™ Serum (U.S.), Standard (Gibco, Fisher Scientific, UK), and stored in a 37 °C CO_2_ incubator. The medium was changed every 48 h to eliminate non-adherent cells. On day 14, the cells that adhered to the cell culture flask and formed colonies were 80–90% confluent. The cells were separated into several cell culture flasks for propagation and passaged using the animal-origin-free TrypLE express enzyme (Gibco, UK) to obtain enough MSCs for further use. Confluent cells in passage 2 were isolated and cryopreserved at a density of 1.5 10^6^/1.8 mL cryovials. They were placed in a pre-master cell bank for quarantine. In this step, the cells were assessed for microbiological sterility test (direct inoculation), mycoplasma assay (culture and PCR according to USP Chapter <63> Mycoplasma Assays), endotoxin assay (referring to USP Chapter <85> Bacterial endotoxin test), and immunophenotyping analysis of CD marker expression (CD73, CD105, CD90, and CD45). The cells were kept in the master and working cell banks once the quality control tests were successful. The cryopreserved vials were thawed 2 weeks before administering the cells. Confirmatory tests were performed by the International Society of Cell Therapy (ISCT), including analyses of cell morphology, cell viability using trypan blue, flow cytometry, MSC multilineage differentiation (into bone, cartilage, and fat), and G-banding karyotyping analysis. As soon as the finished product met the acceptable parameters, it was released and sent to the clinic for injection.

### Intraovarian injection of stem cells

The final product, with a density of 20 × 10^6^ cells/ml, was delivered to the infertility clinic on the day of injection. Under general anaesthesia with midazolam (Caspian Pharmaceutical Co., Iran) and fentanyl (Caspian Pharmaceutical Co., Iran), 150 μl of the prepared solution were injected intravaginally into each patient's ovary. AMH and FSH levels and AFC were checked before cell transplantation. To reduce discrimination, one doctor injected MenSCs into the ovaries. Patients were checked for spontaneous pregnancy 2 months after the injection. When a pregnancy did not happen spontaneously, ICSI was employed.

### Follow-up evaluation

To evaluate the safety and efficacy of MenSCs injections in the enrolled participants, we examined their medical histories, vital signs, blood chemistry, physical examination reports, vaginal sonography, and ovarian function tests at baseline and at 2 months follow-up. At each clinic during pregnancy, pregnancy problems such as miscarriage, ectopic pregnancy, preeclampsia, preterm rupture of membranes, intrauterine foetal mortality, and diabetes were assessed. Throughout pregnancy, patients got the appropriate attention and routine screening testing.

### Ovarian stimulation for ICSI/IVF

Ovarian stimulation of participants was done by the GnRH antagonist protocol. In brief, the contraceptive pill (Aboreihan, Iran) was administered during the cycle preceding ovarian stimulation. Following the injection of recombinant human FSH (GONAL-f®; Merck Serono, Germany, 300 IU) and Human Menopausal Gonadotropin (HMG) (Menotropins®; Karma, Germany, 150 IU), the size of ovarian follicles and their growth rates were evaluated by vaginal ultrasound. GnRH antagonists (Cetrotide®; Merck Serono, Germany) were introduced when the primary follicle grew to 14 mm, and their effects were followed by vaginal ultrasound (Honda 2000–5 MHz, Japan). Every day until at least two follicles were at least 18 mm wide, 0.25 mg was administered subcutaneously. Egg retrieval was performed by vaginal ultrasound 36 h after intramuscular administration of 10,000 IU HCG (Choriomon® (IBSA)). On the third or fifth day following the oocyte retrieval, an embryo transfer (ET) was performed. When an endometrial line wasn't suitable for a patient, embryos were frozen and transplanted during the frozen embryo transfer cycle. Twice daily vaginal administration of Cyclogest 400 mg suppositories (Progesterone PhEur, Actavis, UK) was initiated prior to ET followed by an interval of every 8 h post-embryo transfer for nine weeks.

### Statistical analysis

Analyses were performed by subjects blinded to the study groups. Results are presented as Mean ± Standard Deviation, median, and interquartile range. Qualitative data were expressed as frequencies and percentages. The chi-square test (Fisher's exact test) was used to examine relationships between qualitative variables. Comparisons between groups were made using the Mann–Whitney U test and the Wilcoxon signed-rank test (for nonparametric data). Before and after treatment datasets were evaluated in each group using a paired t test analysis in GraphPad PRISM 6.0. The analysis of one variable at several intervals was performed by a repeated-measures ANOVA test. The Pearson correlation test was used to measure a linear dependence between two variables. *P* ≤ 0.05 was considered statistically significant.

## Results

**Fig. 1:**
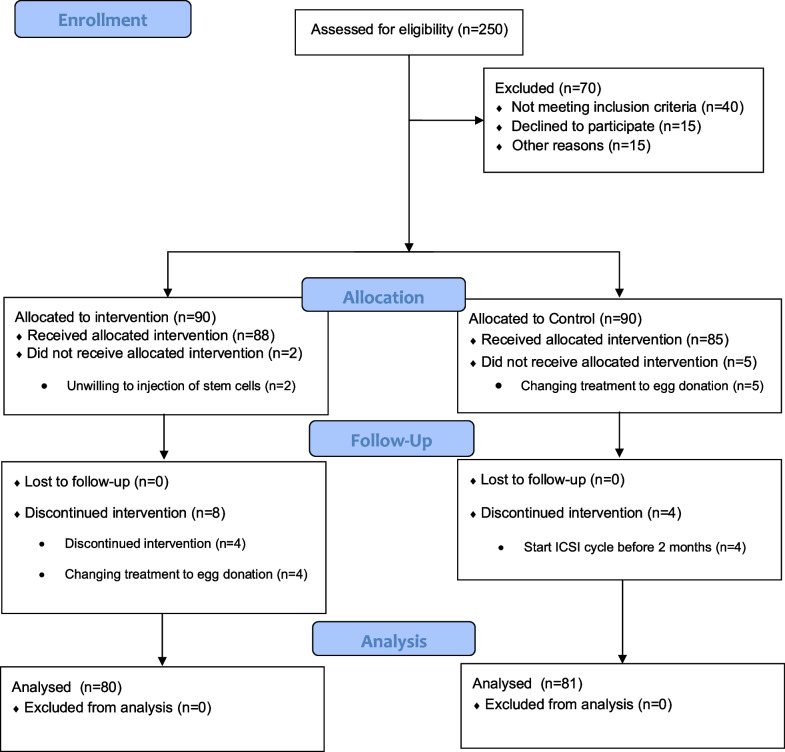
Flow diagram showing enrolment, allocation, follow up and analysis of patients

A total of 250 women were examined for eligibility, and 180 were included in this trial, with 90 assigned to the MenSC-treated group and 90 assigned to the control group for a routine ICSI plan (*N* = 90). Two MenSC patients were disqualified because they refused to inject cells after providing a monthly blood sample. Subsequently, over the course of 2 months post-injection, eight patients opted to discontinue the therapy, with four electing to pursue egg donation and the others declining further treatment. Additionally, after the initial 2-month follow-up, nine patients from the control group were also removed from the research. Five patients changed their treatment plans to receive donor eggs. Four other patients entered the IVF cycle at their request before completing the initial 2-month period (Fig. 1).

Since it has been shown in past research that fertility chances decrease sharply at the age of 40, we performed a post-hoc subgroup age analysis to assess the effect of the age variable on the patients’ response to cell therapy. Table 2 shows that the demographic characteristics, baseline ovarian reserve markers, and previous pregnancies of the women included in the study were similar across treatment arms.

**Table 2:** An analysis of the demographic characteristics of participants in the MenSC therapy and control groups at the onset of the study

Parameters	In ≤ 40years old group			In > 40years old group		
	MenSC group (*n* = 44)	Control group (*n* = 44)	*P*-value	MenSC group (*n* = 44)	Control group (*n* = 41)	*P*-value
Age (years)	34.9 ± 3.6	34.3 ± 3.4	NS	42.7 ± 1.8	42.2 ± 1	NS
duration of infertility (years)	4.8 ± 4	4.9 ± 4.3	NS	25.5 ± 4.8	25 ± 3.5	NS
Gravid number	0.5 ± 0.8	0.3 ± 0.5	NS	0.5 ± 0.6	0.6 ± 0.8	NS
BMI	24.9 ± 3.7	24.9 ± 4	NS	25.5 ± 4.8	25 ± 3.5	NS
Sperm count (mill/ml)	35.5 ± 14.5	33 ± 12.4	NS	29.1 ± 13.4	31.8 ± 16.9	NS
No. of oocytes	1.4 ± 1.4	1.9 ± 1.3	NS	0.8 ± 1.3	1.2 ± 1.2	NS
No. of MII oocytes	0.8 ± 1.1	1.2 ± 1.1	NS	0.4 ± 0.7	0.5 ± 0.9	NS
No. of embryos	0.7 ± 1.1	1 ± 1	NS	0.4 ± 0.7	0.7 ± 0.7	NS
No. of High-quality embryos	0.3 ± 0.7	0.4 ± 0.5	NS	0.09 ± 0.29	0.15 ± 0.3	NS
AMH (ng/mL)	0.4 ± 0.4	0.5 ± 0.4	NS	0.3 ± 0.3	0.4 ± 0.3	NS
FSH (IU/L)	12.8 ± 7.4	11 ± 4.3	NS	16.7 ± 11.4	12.9 ± 5.8	NS
AFC	2 ± 1.8	2.6 ± 1.5	NS	2.0 ± 1.4	2.2 ± 1.0	NS

*BMI*: body mass index; *MII*: metaphase II oocyte; *AMH*: anti-müllerian hormone; *FSH*: follicle-stimulating hormone; *AFC*: antral follicle count; *NS*: not significant; *No.*: number

### Quality control of cultured MenSCs

**Fig. 2:**
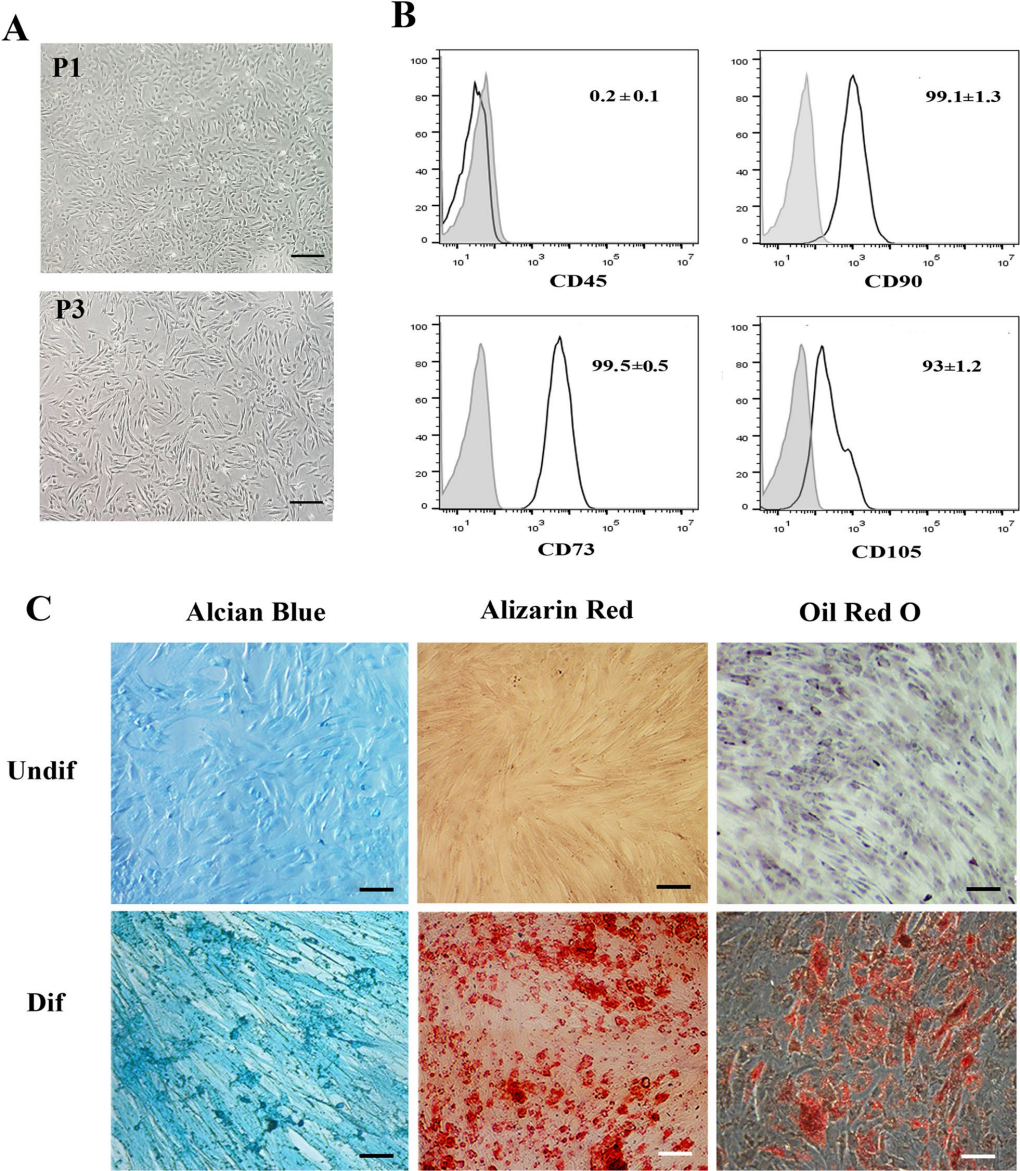
**A.** Morphology of MenSCs at passage 1 and 3. **B.** Representative histograms of MenSCs immunophenotyping by flow cytometry. The cells were negative for CD45. They displayed positive expression for MSCs markers; CD105, CD90, and CD73. **C**. MenSCs differentiation into (1) chondrocytes, (2) osteoblasts, and (3) adipocytes, judged by Alcian blue, alizarin red, and oil red O staining. Scale bar: 100 m

The cultured cells manifested a spindle-shaped fibroblastic morphology. The outcomes derived from the immunophenotyping analysis of the cultured cells were affirmative for CD90 (98.8 ± 1.5%), CD73 (99.2 ± 0.9%), and CD105 (94 ± 2.6%) markers, and negative for the hematopoietic marker CD45 (1.5 ± 1.1%). In addition, a typical karyotype pattern was ascertained, and the absence of microbial growth was observed. The endotoxin samples exhibited no LAL clot formation. Furthermore, DNA amplification analysis on the samples was unconstructive for mycoplasma expression (Fig. 2).

### Adverse events associated with MenSCs injection

No significant adverse reactions, such as allergic reactions, intra-abdominal bleeding, bowel or bladder trauma, or infection, were observed following stem cell therapy. Additionally, no cases of ovarian hyperstimulation syndrome (OHSS) were reported following IVF/ICSI.

### Spontaneous pregnancies in response to cell therapy

**Fig. 3:**
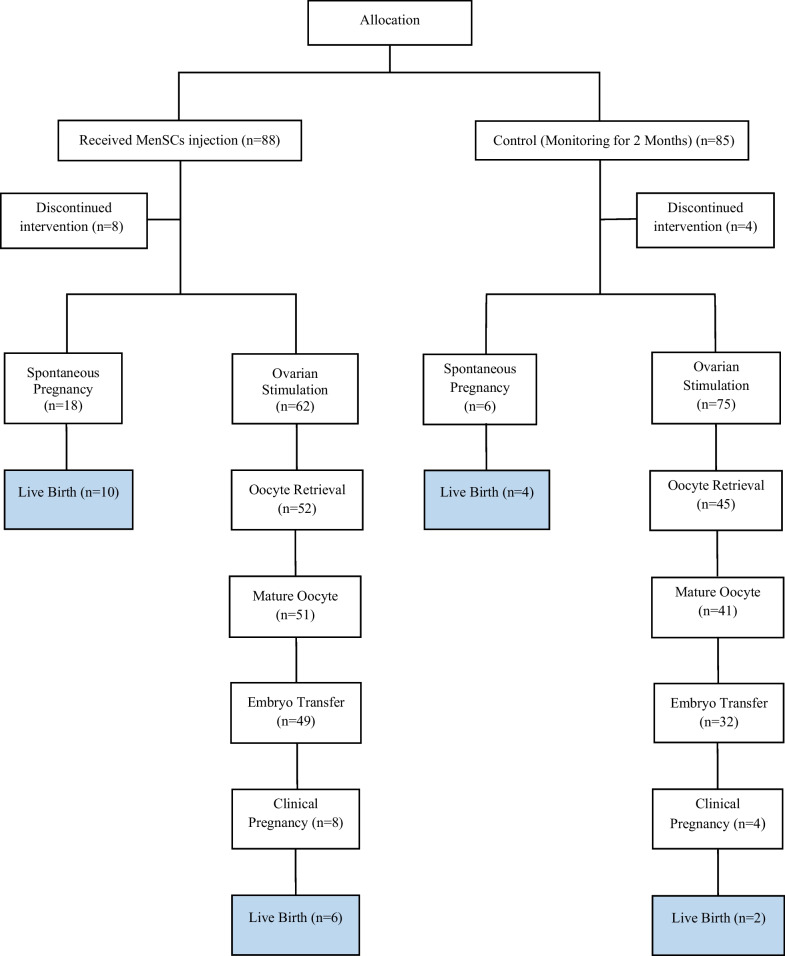
Flowsheet of pregnancy outcomes for all patients

As demonstrated in Fig. 3, following autologous cell delivery, a total of 18 out of 80 individuals (22.5%) became pregnant spontaneously in the cell-treated group, as compared to 6 out of 81 individuals (7.4%) in the control group (*P* = 0.005).

8 of the spontaneously conceived pregnancies were lost as spontaneous miscarriages in the cell-treated group, compared to 2 in the control group (*P* = NS). The remaining pregnancies resulted in timely deliveries. Therefore, 10/18 (55.5%) of spontaneous pregnancies in MenSCs treated resulted in livebirth in comparison to 4/6 (66.6%) in the control group (*P* = NS).

#### Age subgroup analysis

Upon further analysis, it was observed that in the cohort with individuals under the age of 40, there existed a notable difference in the number of spontaneous pregnancies between the treatment and control groups, with a rate of 27.3% versus 9.1%, respectively (*P* = 0.03). However, the live birth rate following spontaneous conception between the two groups showed no significant difference (Table 3).

For patients exceeding the age of 40, the rate of spontaneous pregnancy did not demonstrate a significant discrepancy between the cell-treated group and the control group (16.6% vs. 5.4%) (Table 3). Moreover, despite the fact that the cell-treated group exhibited a higher rate of live births than the control group, this difference was not statistically significant (83.3% vs. 50%).

**Table 3:** Outcomes of the pregnancies in each age subgroup

Parameters	In ≤ 40years old group			In > 40years old group		
	MenSC	Control	*P*-value	MenSC	Control	*P*-value
Spontaneous pregnancy	12/44 (27.3%)	4/44 (9.1%)	0.01	6/36 (16.6%)	2/37 (5.4%)	NS
Pregnancy after ICSI	8/26 (27.6%)	2/14 (4.8%)	0.009	0/23 (0%)	2/18 (5.6%)	NS
Clinical pregnancy	20/44 (50%)	6/44 (13.6%)	< 0.001	6/36 (16.7%)	4/37 (10.8%)	NS
Live birth rate	11/44 (25.0%)	4/44 (9.1%)	0.02	5/36 (13.9%)	2/37 (5.4%)	NS

*ICSI*: intracytoplasmic sperm injection; *NS*: not significant

### Ovarian reserve parameters

**Fig. 4:**
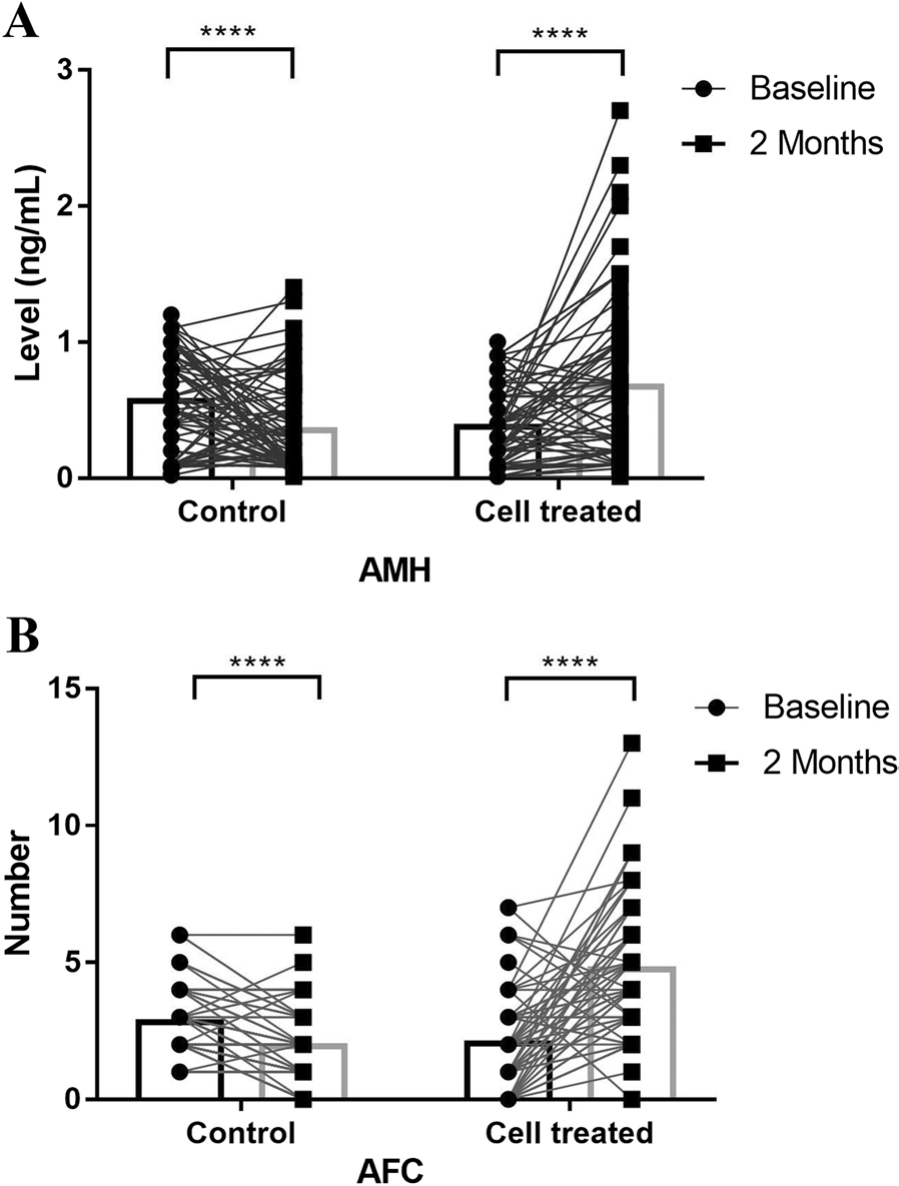
**A**. The MenSCs-treated group exhibited a noteworthy elevation in the level of anti-müllerian hormone (AMH) (*P* < 0.0001), in contrast to the pre-intervention period. Conversely, the control group's AMH levels decreased significantly (*P* < 0.0001) after 2 months. **B.** Subsequent to cell injection, there was a marked augmentation in the antral follicle count (AFC) (*P* < 0.0001), while a substantial decrease was observed in the control group (*P* < 0.0001)

The present study involved the measurement of serum FSH, AMH, and AFC levels before and after intervention in two distinct groups. Our results indicate that the intervention group experienced a statistically significant increase in serum AMH levels (0.8 ± 0.6 vs. 0.4 ± 0.4; *P* < 0.0001), while the control group exhibited a notable decrease in AMH levels (0.3 ± 0.3 vs. 0.6 ± 0.5; *P* < 0.0001) (Fig. 4).

Although the reduction in FSH levels among cell-treated patients did not reach statistical significance, there was a clear trend towards a decrease in hormone levels. Conversely, the control group demonstrated an increase in hormone levels, which was not statistically significant (Table 4). These findings suggest that cell therapy may have the potential to impede the elevation of hormone levels in afflicted patients.

Regarding the AFC, the cell-treated group exhibited a significant 135% increase in the number of antral follicles (4.7 ± 2.4 vs. 2.0 ± 1.6; *P* < 0.0001), while the control group showed declines in the aforementioned measures of 32% (*P* < 0.0001) (Table 4).

**Table 4:** A comparison of ovarian reserve parameters and cycle characteristics before and after treatment

	Stem cell group			Control group		
	Baseline	After 2 months	*P*-value	Baseline	After 2 months	*P*-value
AMH	0.4 ± 0.4	0.8 ± 0.6	< 0.0001	0.6 ± 0.5	0.3 ± 0.3	< 0.0001
FSH	14.8 ± 9.8	13.9 ± 4.3	NS	10.7 ± 5.2	14.3 ± 7.3	NS
AFC	2.0 ± 1.6	4.7 ± 2.4	< 0.0001	2.8 ± 1.2	1.9 ± 1.7	< 0.0001
No. of retrieved oocytes	1.1 ± 1.4	2.9 ± 2.6	< 0.0001	1.2 ± 1.3	0.5 ± 1.1	< 0.0001
No. of MII oocytes	0.6 ± 0.9	2.2 ± 1.9	< 0.0001	1.5 ± 1.3	0.9 ± 1.1	< 0.001
No. of embryos	0.5 ± 1.0	1.8 ± 1.7	< 0.0001	1.2 ± 1.1	0.7 ± 0.9	< 0.0001
No. of high-quality embryos	0.5 ± 0.5	1.3 ± 2.5	< 0.0001	0.6 ± 0.8	0.3 ± 0.6	< 0.002

*AMH*: Anti-Müllerian hormone; *FSH*: follicle-stimulating hormone; *MII*: metaphase II oocyte; No.: number; *NS*: not significant

#### Age subgroup analysis

Post-hoc subgroup analyses using a 40-year age threshold demonstrate that after 2 months of cell injection, ovarian reserve parameters improve significantly compared to before cell therapy in both age groups under 40 years and over 40 years (Table 4). Furthermore, it was discovered that in both aged subgroups, variables improved considerably in response to treatment as compared to the control group (Table 5).

**Table 5:** A comparison of ovarian reserve parameters and cycle characteristics in both age subgroups between cell-treated and control groups

	In ≤ 40years old group			In > 40years old group		
	MenSC	Control	*P*-value	MenSC	Control	*P*-value
AMH	0.85 ± 0.68	0.26 ± 0.22	< 0.001	0.56 ± 0.5	0.32 ± 0.3	0.013
FSH	9.6 ± 6.4	15.4 ± 7.2	< 0.001	17.2 ± 17.5	16 ± 7.2	NS
AFC	5.3 ± 2.6	1.7 ± 1	< 0.001	4.2 ± 2.2	2.2 ± 1	< 0.001
No. of oocytes	4.2 ± 2.7	1 ± 1	< 0.001	1.6 ± 1.5	0.5 ± 1.1	0.002
No. of MII oocytes	3.1 ± 2	0.7 ± 0.9	< 0.001	1.3 ± 1.3	0.4 ± 0.9	< 0.001
No. of embryos	2.4 ± 1.7	0.4 ± 0.6	< 0.001	1.2 ± 1.3	0.3 ± 0.5	< 0.001
No. of high-quality embryos	1.8 ± 1.6	0.1 ± 0.3	< 0.001	0.8 ± 1.2	0.05 ± 0.2	< 0.0001

*AMH*: Anti-Müllerian hormone; *FSH*: follicle-stimulating hormone; *MII*: metaphase II oocyte; *No.*: number; *NS*: not significant

### ICSI/IVF outcomes

As exemplified in Fig. 3, following the deliberate exclusion of patients who experienced spontaneous pregnancy, a total of 62 women in the cell-treated group and 75 women in the control group underwent controlled ovarian hyperstimulation (COH).

Oocyte retrieval was performed on 52 women in the treated group (83.9% of those stimulated), while 30 women in the control group (40%) were unable to undergo oocyte retrieval due to stimulation failure (*P* = 0.02).

It was noted that the average number of oocytes per retrieval before and after stem cell therapy was 1.5 ± 0.2 and 2.6 ± 0.3 (*P* < 0.0001), respectively. In contrast, the control group had 40% fewer oocytes on average after a 2-month follow-up (*P* < 0.0001).

Among the women who underwent oocyte retrieval, 51 women in the treated group (98%) and 41 women in the control group (89.1%) achieved at least one mature oocyte (*P* = 0.005).

In 49 women of the treated group (79% of those stimulated), at least one cleavage-stage embryo was obtained, as compared to 32 women of the control group (42.6% of those stimulated) (*P* < 0.0001).

Among the cell-treated group, 16.3% (8/49) of those who underwent IVF with embryo transfers (ETs) resulted in clinical pregnancies, compared to 12.5% (4/32) of the control group (*P* = NS). Six pregnancies in the treated group and two pregnancies in the control group resulted in live births (*P* = NS). It is worth noting that all born babies were healthy and were followed up for 3 months.

#### Age subgroup analysis

Post-hoc analyses were conducted on subgroups utilizing a 40-year age threshold, which revealed that only the younger cohort exhibited a statistically significant increase in pregnancy and live birth rates subsequent to undergoing ICSI/IVF (*P* = 0.009 and *P* = 0.02, respectively). However, in the age group above 40 years, although there was a noticeable enhancement in ovarian reserve indicators, no substantial variance in the number of pregnancies resulting from ICSI/IVF or the quantity of live births compared to the control group was found. A comprehensive breakdown of the data is presented in Table 3.

### Correlation between ovarian reserve marker and ICSI/IVF outcomes

**Fig. 5:**
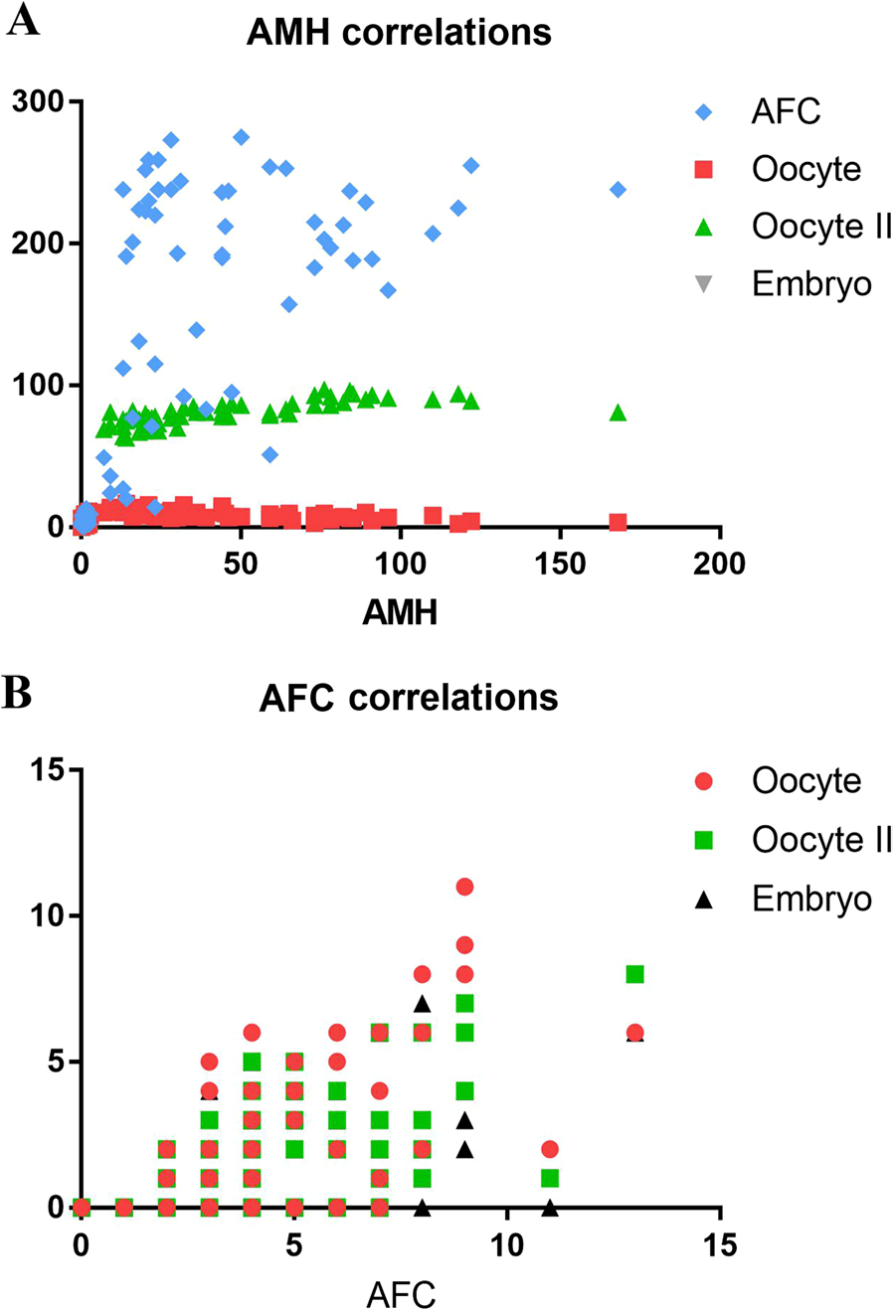
**A.** There are positive correlations between the level of anti-müllerian hormone (AMH) and controlled ovarian hyperstimulation outcomes in the cell-treated group. **B.** Graph showing significant positive correlations between the level of antral follicular count (AFC) and controlled ovarian hyperstimulation outcomes in the cell-treated group

The present study aimed to analyse the correlation between ovarian reserve markers and COH outcomes in the group that received cellular treatment (Table 6). The results demonstrated statistically significant positive correlations between AMH levels and the number of retrieved oocytes, the number of mature oocytes, and the number of embryos (*P* = 0.006, *P* < 0.0001, and *P* = 0.003, respectively). Additionally, similar positive associations were observed between AFC and the number of retrieved oocytes, the number of mature oocytes, and the number of embryos (*P* < 0.0001, *P* < 0.0001, and *P* = 0.0013, respectively). Specifically, patients who exhibited higher levels of AMH and AFC after cellular treatment demonstrated a better response to COH (Fig. 5).

**Table 6:** There are positive correlations between the levels of anti-müllerian hormone (AMH) and antral follicular count (AFC) and controlled ovarian hyperstimulation outcomes in the cell-treated group

Parameters	Oocyte count	*P* value	MII oocyte count	*P* value	Embryo count	*P* value
AMH	*r* = 0.25	0.006	*r* = 0.7569	< 0.0001	*r* = 0.3805	0.003
AFC	*r* = 0.616	< 0.0001	*r* = 0.5753	< 0.0001	*r* = 0.3982	0.0013

*AMH*: Anti-Müllerian hormone; *AFC*: antral follicle count; *MII*: metaphase II oocyte

## Discussion

The current non-randomized controlled clinical study endeavoured to evaluate the impact of a new MenSC-based treatment on parameters of ovarian reserve as well as pregnancy outcomes in females suffering from POR. In the third stage of this study, akin to the preceding phases, our findings represent the successful implementation of autologous MenSC as a viable treatment option for women afflicted with POR, resulting in enhanced ovarian insufficiency. Notably, this therapeutic modality is associated with a marked amplification of ovarian reserve parameters, a substantial increase in oocyte quantity and quality, heightened fertilization rates, ameliorated embryo quality, an elevated pregnancy rate, and a superior live birth rate compared to the control group.

Overcoming the challenge of achieving conception in patients with POR through the use of their eggs has been a significant hurdle for fertility experts, particularly after several IVF cycles that are often terminated due to insufficient or no ovarian response [37]. For years, researchers have been striving to increase the likelihood of successful pregnancy in women with POR by augmenting the quality and quantity of eggs and embryos. Despite efforts to enhance ovulation stimulation cycles [38, 39], and utilize growth hormones [40–42], testosterone [43], and DHEA [44, 45] during ovulation treatment cycles, none of the proposed methods have yielded satisfactory outcomes to date. However, a glimmer of hope for POR patients emerged in 2018 with the revelation by Herraiz and colleagues of the potential of stem cells for ovarian regeneration [20]. In this pilot study, consisting of 17 women with POR, BMSCs were administered directly into the ovarian artery through intra-arterial catheterization on one side, while the contralateral ovary served as a control. The authors reported a significant increase in serum AMH levels in a subset of patients within the first four weeks of follow-up, with an improvement in AFC observed two weeks after initiating treatment. Our investigations corroborated these findings, with a statistically significant elevation in serum AMH levels and antral follicle count 2 months following MenSC injection, whereas the control group experienced significant reductions. Although not significant, our patients in the cell-treated group exhibited a decrease in blood FSH levels compared to baseline, illustrating the beneficial effect of MenSCs on ovarian function relative to the control group, which demonstrated a significant rise in hormone levels.

AMH is produced by the granulosa cells of pre-antral and small antral follicles, with levels gradually increasing from the secondary stage onward until the small antral follicle stage [46]. Conversely, AFC is known as an indicator of the number of follicles that will undergo maturation during the ovulation treatment cycle [47]. Therefore, the levels of AMH and AFC are valuable markers for assessing ovarian reserve function in POR patients [48]. The data obtained from prior clinical investigations conducted on individuals diagnosed with POF [29, 35] and POR [20], as well as Phase I and II of the current study [36], consistently demonstrated that the intraovarian injection of MenSC led to a significant elevation in the level of serum AMH and AFC when compared to pre-treatment levels. The present study reveals a favourable AFC response in patients with POR to the 2-month treatment, indicating that the primary beneficiaries of cell therapy were likely the secondary follicles. Furthermore, previous studies have demonstrated the expeditious growth of secondary follicles, leading to the production of preovulatory follicles in a few weeks upon the implementation of in vitro activation and ovarian fragmentation procedures in patients with POI and animal models [49–51]. On the contrary, recent investigations have demonstrated a positive correlation between serum AMH levels and AFC with the number of retrieved oocytes. Clinical studies conducted by Kotanidi et al. uncovered that women with more retrieved oocytes and suitable supplementary embryos for cryopreservation exhibit higher levels of AMH and numbers of AFC [52, 53]. Herraiz et al. have also reported that the administration of BMSC has resulted in an increased number of antral follicles and retrieved oocytes, leading to an improvement in reproductive outcomes compared to prior stem cell administration [20]. Our observations validate these findings, as our data provide significant evidence that the utilization of MenSCs in the IVF phase can considerably reduce the cycle cancellation rate by augmenting the number of antral follicles and retrieved oocytes, as well as inducing a remarkable enhancement in oocyte and embryo quality.

The age of a woman is one of the most significant determinants affecting the chances of conception, whether natural or through ART [54]. Hanoch et al. conducted a study to evaluate the differences in pregnancy rates between young and older patients with low ovarian response [55]. They reported a significantly higher clinical pregnancy rate in the younger group. In line with previous research, our further analyses of age subgroups showed a significant enhancement in ovarian reserve markers in the cell therapy group, resulting in a higher likelihood of pregnancy, both naturally and following IVF, but only in under-40 individuals compared to the control group. Specifically, only the younger cell therapy group exhibited a significant decrease in abortion rates, resulting in a significantly higher live birth rate. These data reveal that although older patients with low ovarian response benefit from stem cell therapy by improving ovarian reserve markers, the positive effects of stem cells on the restoration of the ovarian microenvironment are not enough to result in successful pregnancies.

Despite the encouraging findings of both animal and human studies on the efficacy of MSCs in treating reproductive diseases [56–59], the mechanisms by which these MSCs enhance pregnancy and live birth rates have yet to be fully understood. Poor responders, who typically have a low count of stimulus-responsive antral follicles, may not necessarily be completely lacking in small preantral follicles [60]. However, a damaged ovarian niche may be unable to sustain the efficient physiological requirements for rapid proliferation and differentiation of granulosa cells [61]. Additionally, the vascularization impairment in these patients might contribute to their absence of ovarian response [21]. Infusion of BMDSC has been shown to enhance local vascularization in chemotherapy-induced POR and POI conditions [19, 21]. In addition, previous studies indicate that transplantation of BMSCs has the potential to hinder apoptosis of ovarian granulosa cells and enhance folliculogenesis in animal models of PCOS and POF through anti-inflammatory, anti-oxidative, and anti-apoptotic pathways [62, 63]. Similar results have been obtained in animal studies using adipose-derived stem cells (ADSC), which have been shown to restore ovarian function and promote oocyte quality, embryonic development, and fertility in animal models of POI and PCOS [64, 65]. Furthermore, amniotic fluid stem cells (AFSC) have been found to restore reproductive ability by preserving healthy follicles and preventing follicular atresia in an ovarian model of chemotherapy-induced POF [66]. Regarding MenSCs, existing studies indicate that they are capable of releasing several growth factors and cytokines, such as vascular endothelial growth factor (VEGF), hepatocyte growth factor (HGF), and IGF-1, which could potentially enhance ovarian function and restore the ovarian niche to sustain its current follicular pool [67, 68]. Previous animal studies have shown that MenSCs produce higher levels of ovarian markers, increased ovarian weight, higher plasma estradiol (E2) levels, and a normal number of follicles after being transplanted into mice [14, 69]. Furthermore, MenSCs have been observed to reduce apoptosis in granulosa cells and the fibrosis of the ovarian interstitium, increase the number of follicles, and improve the ovary's microenvironment through the release of FGF2 [56]. Additionally, the transplantation of MenSCs into the ovary may stimulate the development of very small embryonic-like stem cells and ovarian stem cells as, two potential populations of stem cells in adult mammalian ovaries [70]. Although there is no evidence to support the plausibility of injected MenSCs differentiating into a range of ovarian cells such as theca cells, granulosa cells, and vascular endothelial cells, this hypothesis is based on research demonstrating that bipotent mesenchymal progenitors can generate oocytes, granulosa cells, and follicles in the tunica albuginea of adult human ovaries [71]. In the current study, taking into account the occurrence of the restoration of ovarian function resulting in natural pregnancy within a mere 2 months following the administration of MenSCs, it appears that the latter event is less feasible. In fact, it is more probable that the dual paracrine effect of MenSCs in both the ovarian niche and the maturation of follicles, as well as the improvement of their communication, are more implicated in this phenomenon. While these findings provide promising initial evidence for the potential application of MenSCs in the treatment of infertility caused by POR, further investigation is imperative to ascertain their safety and efficacy in clinical contexts.

The current study, which builds upon previous phases, has demonstrated the safety and effectiveness of cell therapy utilizing MenSCs to address infertility concerns among a larger population of females with POR. As a result, a new methodology has been developed to increase the likelihood of natural conception and successful delivery for POR females. For those who cannot avoid ovarian stimulation and ICSI, MenSC therapy may offer advantages in terms of oocyte quantity and quality, oocyte fertilization rate, and embryo quality. Our prospective study, which spanned one year, had the key advantage of closely monitoring female participants from enrolment to delivery. Additionally, the comparison between MenSCs and control groups in two distinct age cohorts with similar baseline demographic features allowed for an accurate evaluation of MenSCs' effectiveness. However, a major limitation of this study was its lack of randomization. Women self-selected which study group to join after being informed about MenSCs' utility in regenerative medicine, which may have resulted in selection bias. Those in better socio-economic positions may have chosen the self-paid intervention instead of the control arm. The study's small sample size also restricts its scope, potentially limiting its ability to detect meaningful effects on pregnancy outcomes such as clinical pregnancy rate, miscarriage rate, and live birth rate.

Nevertheless, akin to other comparable investigations, this clinical study encountered several challenges. The process of transforming cells into efficacious, secure, and cost-effective therapies is a meticulous and intricate endeavour, even after identifying all fundamental parameters. The production of living-cell products necessitates aseptic processing and transportation, frequently with a limited shelf-life. Consequently, to ensure compliance with GMP guidelines for validation and quality control of the final cell product, we strived to adhere to regulatory provisions established by the Food and Drug Administration (FDA) and European Medicines Agency (EMA). In this manner, our primary focus was on the safety, efficacy, and consistency of the cell products. Moreover, there exist potential hazards associated with MSC application, particularly in specific cellular microenvironments, which demand careful consideration in long-term monitoring and follow-up assessments. Hence, further randomized parallel studies with a larger sample size and an extended follow-up period are indispensable to definitively establish the impact of MenSCs on ovarian function and live birth rate, thereby validating the applicability of our findings in a clinical context.

## Conclusion

In conclusion, it may be deemed appropriate to consider the administration of autologous MenSCs via intraovarian injection in females experiencing POR. It is suggested that individuals under the age of 40 years old would be the most suitable candidates for this approach, as it could potentially enhance pre-existing ovarian reserve and improve ICSI/IVF outcomes. However, in order to broaden the scope of clinical implementation, several queries will need to be addressed through prospective randomized clinical trials. These include, but are not limited to, determining the duration of stem cell efficacy, identifying more appropriate clinical cases for this therapy, and validating the appropriate dose of MenSCs.

## Data Availability

All of the data generated and analysed during this study are included in our manuscript.

## Abbreviations

ART: Assisted reproductive technology
POR: Poor ovarian response
AFC: Antral follicular count
AMH: Anti-Müllerian hormone
IVF: In vitro fertilization
MSC: Mesenchymal stromal cell
POF: Premature ovarian failure
IGF-1: Insulin-like growth factor I
TGF-ß: Transforming growth factor ß
FGF1: Fibroblast growth factor 1
FGF2: Fibroblast growth factor 2
EGF: Epidermal growth factor
VEGF: Vascular endothelial growth factor
HGF: Hepatocyte growth factor
BMSC: Bone marrow-derived mesenchymal stem cells
MenSCs: Menstrual blood-derived stromal cells
AST: Aspartate aminotransferase
ALT: Alanine aminotransferase
BUN: Blood urea nitrogen
HIV: Human immunodeficiency virus
HCV: Hepatitis C virus
HBV: Hepatitis B virus
VDRL: Venereal disease research laboratory
PBS: Phosphate-buffered saline
EDTA: Ethylene diamine tetra acetic acid
DMEM-F12: Dulbecco Modified Eagle Medium-F12
PCR: Polymerase chain reaction
CD: Cluster of differentiation
GnRH: Gonadotropin-releasing hormone
FSH: Follicle-stimulating hormone
HMG: Human menopausal gonadotropin
GCP: Good clinical practice
GMP: Good manufacture practice
